# Supplementary figures and images for: Inferring personal intake recommendations of phosphorous and potassium for end-stage renal failure patients by simulating with Bayesian hierarchical multivariate model
Source: PLoS One. 2024 Feb 6;19(2):e0291153. doi: 10.1371/journal.pone.0291153 (PMC10846746; doi:10.1371/journal.pone.0291153)

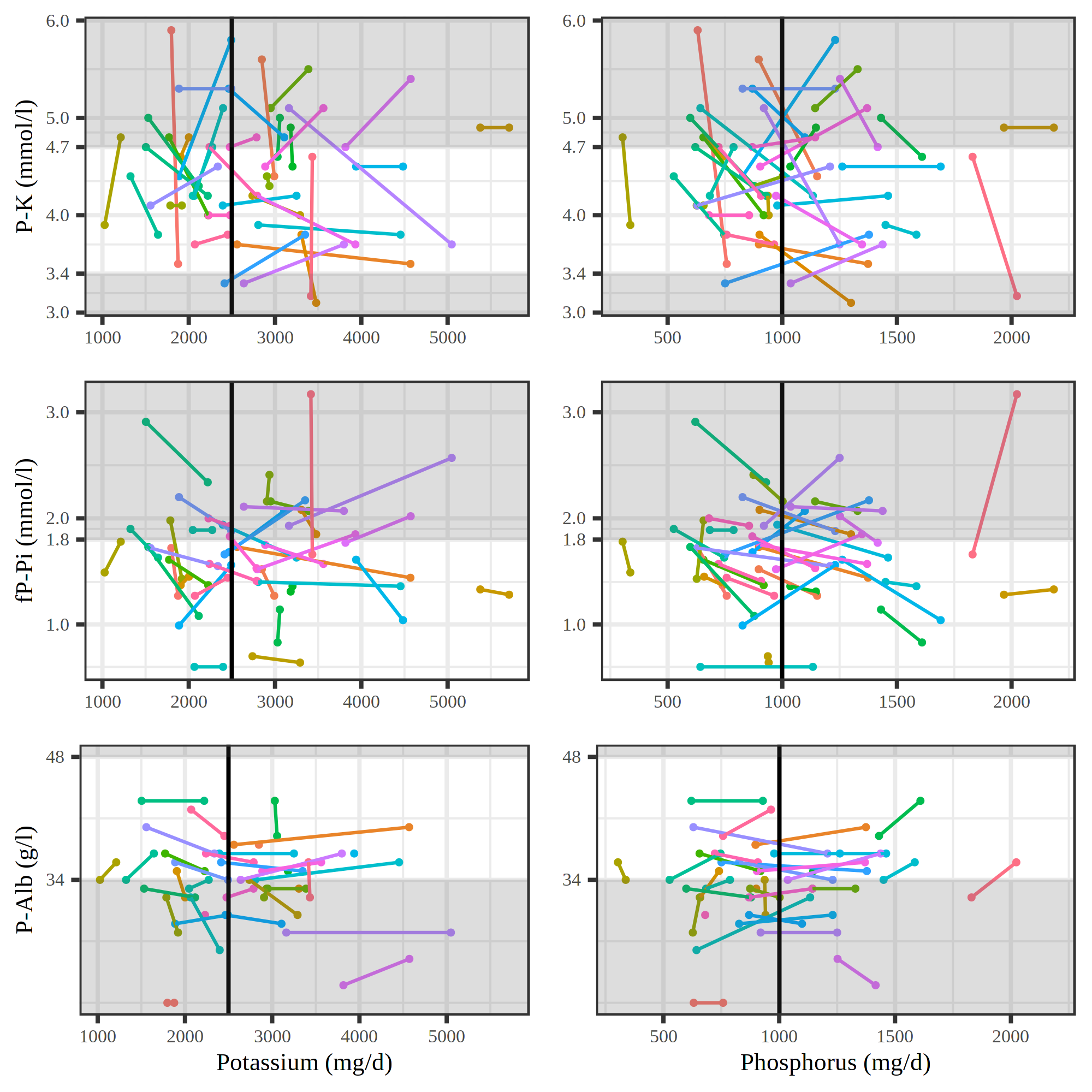

Supplement: S1 Fig — The recommended normal ranges are denoted with white areas. (TIF) [file pone.0291153.s001.tif]

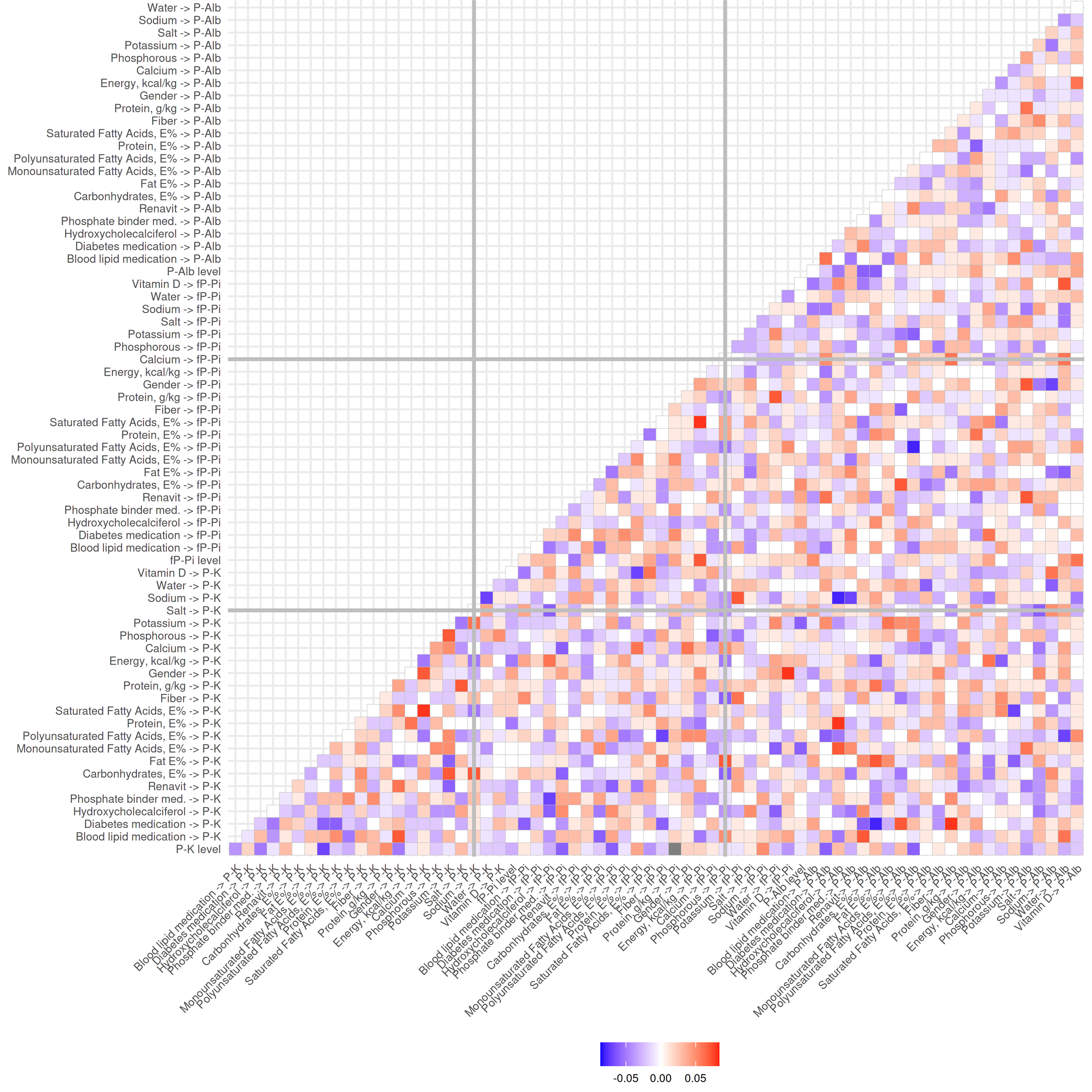

Supplement: S2 Fig — The figure is plotted with ggcorrplot package for R language (v 0.1.4, https://cran.r-project.org/web/packages/ggcorrplot). (TIF) [file pone.0291153.s002.tif]

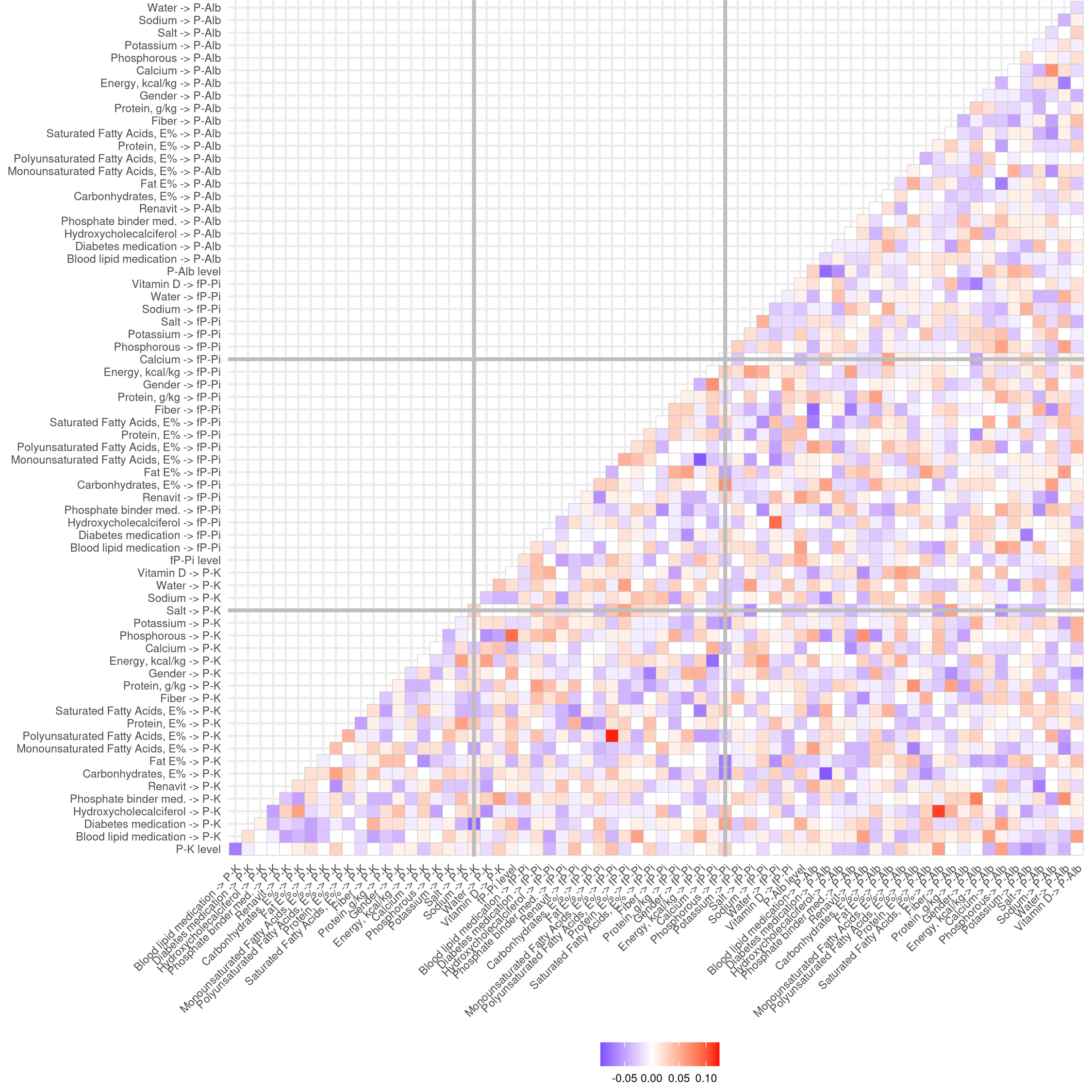

Supplement: S3 Fig — The figure is plotted with ggcorrplot package for R language (v 0.1.4, https://cran.r-project.org/web/packages/ggcorrplot). (TIF) [file pone.0291153.s003.tif]

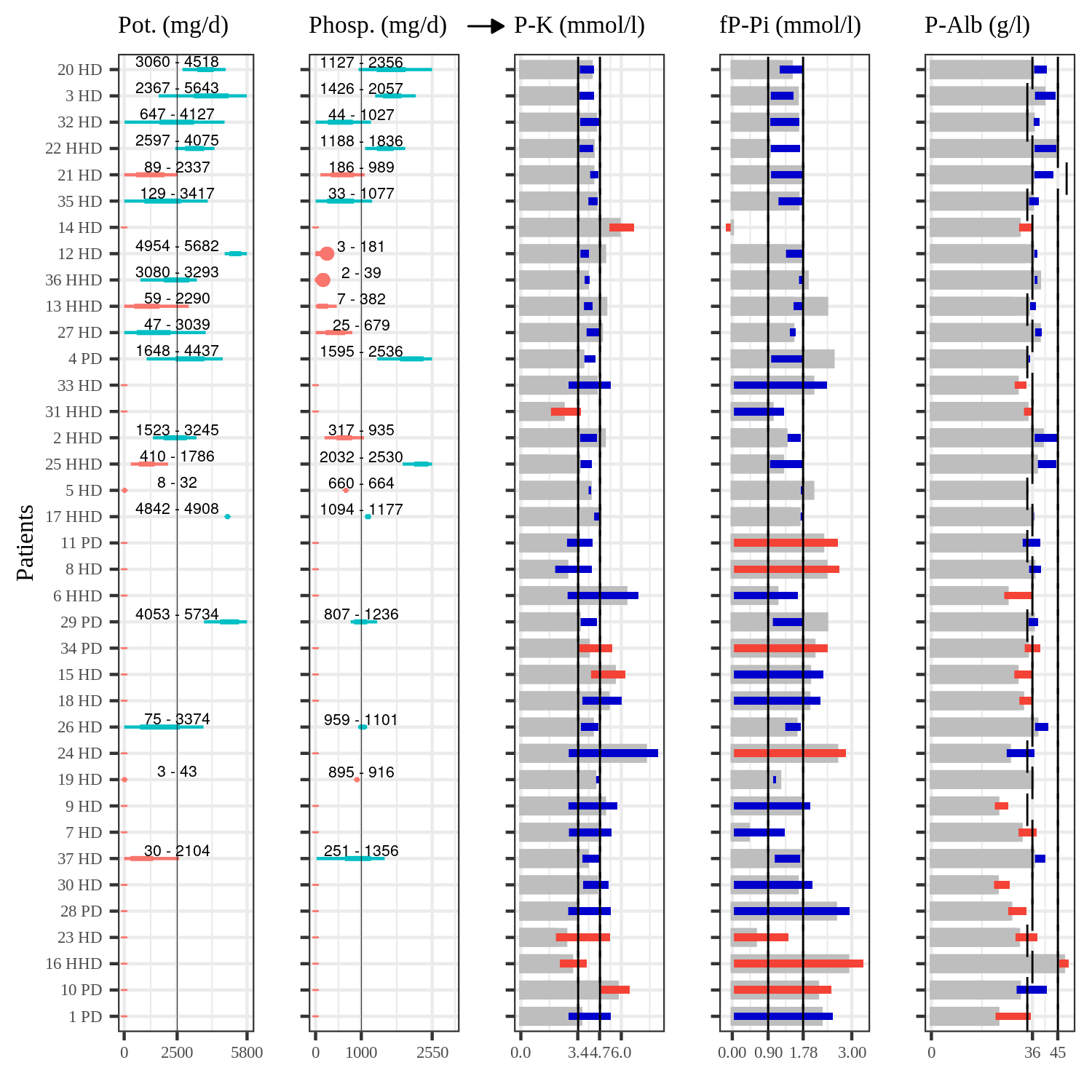

Supplement: S4 Fig — The figure is plotted with ggplot2 package for R language (v 3.4.1, https://ggplot2.tidyverse.org). (TIF) [file pone.0291153.s004.tif]

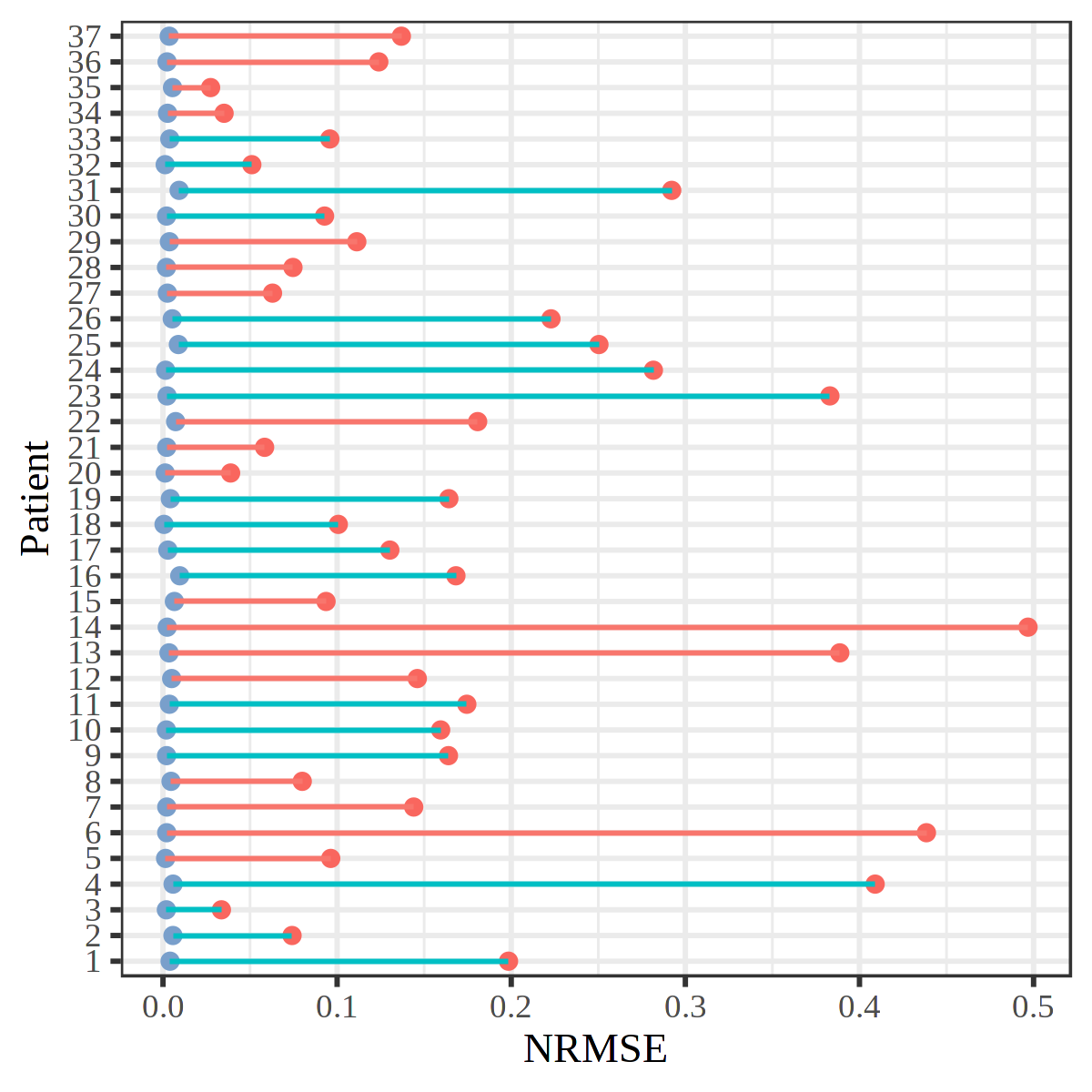

Supplement: S5 Fig — Blue points indicate the in-sample error and red points indicate the error from cross-validation prediction. The lines between the points highlight the increased error between in-sample modeling and prediction. Alternating colors of the lines denote the folds of the cross-validation. The figure is plotted with ggplot2 package for R language (v 3.4.1, https://ggplot2.tidyverse.org). (TIF) [file pone.0291153.s005.tif]
