# Supplementary material for: Inferring personal intake recommendations of phosphorous and potassium for end-stage renal failure patients by simulating with Bayesian hierarchical multivariate model
Source: PLoS One. 2024 Feb 6;19(2):e0291153. doi: 10.1371/journal.pone.0291153 (PMC10846746; doi:10.1371/journal.pone.0291153)
Supplement: S1 Table — This structure of correlations is used in estimating the personal effects based on personal intake and matching concentrations. (PDF) [file pone.0291153.s006.pdf]

Table S1: Strongest correlations between all treatment effects and treatment effects of potassium and phosphorous. This estimated structure of correlations is used to infer the treatment effects based on the intake and the corresponding concentrations.

Treatment-level effects of nutrients		
**Effect 1**	**Effect 2**	**Correlation**
Renavit → fP-Pi	Potassium→ P-Alb	0.074
Gender → fP-Pi	Potassium→ P-Alb	0.071
Sodium → P-K	Potassium → fP-Pi	0.065
Protein, g/kg → P-Alb	Potassium→ P-Alb	0.063
Sodium → P-K	Potassium → P-K	0.058
Calcium → P-K	Potassium → P-K	0.053
MUFA, E% → P-K	Potassium → P-K	0.052
PUFA, E% → P-Alb	Potassium → P-K	0.050
Diabetes medication → P-Alb	Potassium → P-K	0.047
Vitamin D → fP-Pi	Potassium→ P-Alb	0.045
Blood lipid medication → fP-Pi	Potassium → fP-Pi	0.044
Phosphorous → P-Alb	Potassium→ P-Alb	0.043
PUFA, E% → fP-Pi	Potassium → P-K	-0.042
Salt → P-K	Potassium→ P-Alb	-0.043
Hydroxycholecalciferol → P-K	Potassium → P-K	-0.045
Sodium → fP-Pi	Potassium→ P-Alb	-0.048
Sodium → P-Alb	Potassium→ P-Alb	-0.049
MUFA, E% → P-Alb	Potassium→ P-Alb	-0.051
P-Alb level	Potassium → P-K	-0.056
PUFA, E% → P-Alb	Potassium → fP-Pi	-0.059
Potassium → P-K	Phosphorous → P-K	0.066
Blood lipid medication → P-K	Phosphorous → fP-Pi	0.054
Calcium → P-K	Phosphorous → fP-Pi	0.051
MUFA, E% → P-K	Phosphorous → P-K	0.050
Protein, E% → fP-Pi	Phosphorous → fP-Pi	0.044
Salt → fP-Pi	Phosphorous→ P-Alb	0.043
PUFA, E% → fP-Pi	Phosphorous→ P-Alb	0.042
Calcium → P-K	Phosphorous → P-K	0.038
SFA, E% → fP-Pi	Phosphorous → fP-Pi	0.036
Hydroxycholecalciferol → P-K	Phosphorous → fP-Pi	-0.037
SFA, E% → fP-Pi	Phosphorous → P-K	-0.037
Carbonhydrates, E% → P-K	Phosphorous → P-K	-0.039
Phosphorous → fP-Pi	Phosphorous→ P-Alb	-0.039
Salt → P-Alb	Phosphorous → fP-Pi	-0.039
Carbonhydrates, E% → fP-Pi	Phosphorous → P-K	-0.040
Phosphate binder med. → P-Alb	Phosphorous → fP-Pi	-0.042
PUFA, E% → fP-Pi	Phosphorous → fP-Pi	-0.048
SFA, E% → P-Alb	Phosphorous → fP-Pi	-0.053
Carbonhydrates, E% → P-K	Phosphorous → fP-Pi	-0.060
Calcium → fP-Pi	Phosphorous → P-K	-0.063
