# Supplementary material for: Inferring personal intake recommendations of phosphorous and potassium for end-stage renal failure patients by simulating with Bayesian hierarchical multivariate model
Source: PLoS One. 2024 Feb 6;19(2):e0291153. doi: 10.1371/journal.pone.0291153 (PMC10846746; doi:10.1371/journal.pone.0291153)
Supplement: S2 Table — This structure of correlations is used in estimating the personal effects based on personal intake and matching concentrations. (PDF) [file pone.0291153.s007.pdf]

Table S2: Strongest correlations between all personal effects and effects of potassium and phosphorous. This estimated structure of correlations is used to infer the personal effects based on the intake and the corresponding concentrations.

Personal effects of nutrients		
**Effect 1**	**Effect 2**	**Correlation**
Sodium -> fP-Pi	Potassium-> P-Alb	0.049
Gender -> P-Alb	Potassium -> P-K	0.046
Fiber -> P-K	Potassium -> fP-Pi	0.044
PUFA, E% -> P-K	Potassium -> P-K	0.043
P-Alb level	Potassium-> P-Alb	0.041
Sodium -> P-Alb	Potassium -> fP-Pi	0.037
Blood lipid medication -> P-K	Potassium -> P-K	0.035
Salt -> P-Alb	Potassium -> P-K	0.034
P-Alb level	Potassium -> fP-Pi	0.032
SFA, E% -> P-K	Potassium-> P-Alb	0.031
Water -> P-K	Potassium -> fP-Pi	-0.030
Water -> fP-Pi	Potassium -> fP-Pi	-0.030
Hydroxycholecalciferol -> fP-Pi	Potassium -> fP-Pi	-0.031
MUFA, E% -> P-K	Potassium -> P-K	-0.033
Phosphorous -> P-K	Potassium -> P-K	-0.035
Salt -> P-K	Potassium-> P-Alb	-0.040
Diabetes medication -> P-Alb	Potassium-> P-Alb	-0.040
Water -> P-Alb	Potassium -> P-K	-0.046
Calcium -> fP-Pi	Potassium -> P-K	-0.051
Phosphorous -> fP-Pi	Potassium -> P-K	-0.058
fP-Pi level	Phosphorous -> P-K	0.088
Renavit -> P-Alb	Phosphorous -> P-K	0.070
Diabetes medication -> P-K	Phosphorous -> fP-Pi	0.056
Hydroxycholecalciferol -> fP-Pi	Phosphorous -> P-K	0.053
SFA, E% -> fP-Pi	Phosphorous-> P-Alb	0.051
Vitamin D -> P-Alb	Phosphorous -> P-K	0.051
Protein, g/kg -> fP-Pi	Phosphorous -> P-K	0.049
Hydroxycholecalciferol -> P-K	Phosphorous -> fP-Pi	0.041
Diabetes medication -> fP-Pi	Phosphorous -> P-K	0.039
MUFA, E% -> P-Alb	Phosphorous -> P-K	0.036
P-K level	Phosphorous-> P-Alb	-0.037
Blood lipid medication -> P-Alb	Phosphorous-> P-Alb	-0.037
MUFA, E% -> P-K	Phosphorous-> P-Alb	-0.039
Calcium -> P-K	Phosphorous -> fP-Pi	-0.044
Hydroxycholecalciferol -> P-K	Phosphorous -> P-K	-0.050
Fiber -> P-Alb	Phosphorous-> P-Alb	-0.051
Fat E% -> fP-Pi	Phosphorous -> fP-Pi	-0.053
Water -> P-K	Phosphorous -> P-K	-0.055
Diabetes medication -> P-Alb	Phosphorous -> P-K	-0.058
Carbonhydrates, E% -> P-K	Phosphorous -> fP-Pi	-0.069
