# Supplementary material for: Inferring personal intake recommendations of phosphorous and potassium for end-stage renal failure patients by simulating with Bayesian hierarchical multivariate model
Source: PLoS One. 2024 Feb 6;19(2):e0291153. doi: 10.1371/journal.pone.0291153 (PMC10846746; doi:10.1371/journal.pone.0291153)
Supplement: S3 Table — Nutrition effect magnitudes from nutrients and other modeled features (j = 1, …, 22) to blood concentrations (i = 1, …, 3) for analyzed patients (p = 1, …, 37) in all three additive levels of the model. General effects (β^ij) are shown to vary between patients in home hemodialysis, hospital hemodialysis, and peritoneal dialysis. The first column of each dialysis type (avg) shows the typical effect of the treatment (β^ij+g^ijk,k=1,…,3) that can further vary personally. Minimum and maximum of these personal effects are shown within each treatment (β^ij+g^ijk+b^ijp). The table is sorted in decreasing order of between-treatment variation (σ^g) and all the estimates include their 90%-credible intervals. (PDF) [file pone.0291153.s008.pdf]

Nutrient	Conc.	General effect	Home hemodialysis			Hospital hemodialysis			Peritoneal dialysis		
			**avg**	**min**	**max**	**avg**	**min**	**max**	**avg**	**min**	**max**
Water	P-Alb	-2.04 [−6.16; 2.97]	**-2.07** [−5.76; 1.76]	-2.60 [−8.07; 2.01]	-2.06 [−6.13; 2.47]	**-0.23** [−3.21; 2.52]	-0.60 [−4.28; 2.84]	0.11 [−3.60; 3.77]	**-1.19** [−5.93; 4.22]	-1.53 [−6.39; 4.00]	-1.12 [−5.86; 4.42]
Blood lipid medication	P-Alb	0.36 [−6.33; 7.76]	**1.25** [−6.00; 10.22]	0.24 [−8.77; 11.01]	1.97 [−6.60; 12.07]	**0.14** [−5.39; 5.61]	-1.17 [−7.39; 5.64]	1.19 [−4.35; 6.96]	**1.14** [−5.92; 10.13]	0.60 [−8.54; 9.56]	1.81 [−5.82; 11.07]
Hydroxycholecalciferol	P-K	0.50 [−3.57; 3.68]	**-0.42** [−5.08; 2.69]	-0.52 [−5.31; 2.40]	-0.19 [−5.07; 2.73]	**-0.51** [−2.17; 1.19]	-0.82 [−2.69; 1.19]	-0.25 [−2.28; 2.02]	**-0.94** [−3.42; 1.72]	-1.08 [−3.85; 1.76]	-0.83 [−3.48; 1.84]
Calcium	P-Alb	-0.08 [−10.35; 6.03]	**1.85** [−2.93; 7.46]	1.22 [−3.36; 6.27]	2.25 [−3.61; 9.68]	**0.88** [−3.47; 4.95]	-0.32 [−5.36; 4.17]	1.59 [−3.38; 6.13]	**1.64** [−2.90; 6.84]	2.03 [−3.76; 8.83]	2.60 [−3.21; 11.67]
Sodium	P-Alb	1.22 [−8.68; 15.01]	**2.23** [−5.69; 12.83]	2.00 [−6.13; 11.84]	2.80 [−5.80; 15.93]	**-0.87** [−6.51; 5.45]	-1.39 [−7.65; 5.06]	-0.28 [−6.33; 7.10]	**-3.02** [−13.00; 7.48]	-3.28 [−13.36; 7.35]	-2.60 [−12.82; 8.02]
Salt	P-Alb	1.60 [−8.59; 10.81]	**3.76** [−7.68; 12.53]	3.28 [−8.23; 11.96]	4.52 [−6.23; 12.73]	**1.82** [−3.68; 6.93]	1.27 [−4.49; 6.56]	2.27 [−4.09; 9.07]	**-0.39** [−11.05; 8.80]	-0.81 [−12.25; 8.04]	-0.31 [−11.56; 8.59]
Gender	P-Alb	1.67 [−5.48; 10.14]	**0.36** [−6.76; 6.92]	-0.53 [−11.15; 7.42]	1.69 [−8.20; 10.28]	**2.73** [−3.79; 9.68]	1.43 [−7.72; 9.22]	4.13 [−3.80; 11.67]	**1.64** [−7.19; 9.96]	0.77 [−11.13; 10.90]	2.73 [−7.01; 13.42]
Phosphate binder med.	P-Alb	-2.78 [−9.87; 4.87]	**-2.68** [−10.50; 7.83]	-4.26 [−18.77; 8.61]	-1.59 [−9.86; 8.98]	**-1.95** [−9.93; 6.69]	-4.25 [−16.99; 7.20]	-0.96 [−11.15; 10.81]	**-0.37** [−8.93; 8.19]	-1.32 [−10.67; 7.77]	0.84 [−10.26; 12.01]
Energy, kcal/kg	P-Alb	1.60 [−11.49; 15.39]	**-0.40** [−12.86; 13.00]	-1.39 [−13.20; 11.42]	0.69 [−12.25; 15.89]	**-1.40** [−12.93; 9.31]	-2.93 [−14.33; 8.17]	-0.18 [−12.79; 11.23]	**1.06** [−11.93; 16.56]	-0.18 [−13.30; 15.81]	2.15 [−12.02; 18.26]
Carbohydrates, E%	P-Alb	1.29 [−14.77; 15.35]	**2.67** [−13.38; 18.00]	2.28 [−13.04; 17.08]	3.71 [−11.99; 18.43]	**1.80** [−13.38; 15.62]	0.91 [−15.21; 15.17]	2.33 [−11.97; 15.69]	**0.82** [−15.89; 15.37]	0.16 [−16.99; 15.88]	1.33 [−15.87; 15.52]
Monounsaturated Fatty Acids, E%	P-Alb	1.81 [−6.77; 13.45]	**2.82** [−3.72; 12.69]	1.80 [−5.70; 12.61]	3.92 [−3.14; 13.42]	**1.39** [−4.01; 6.82]	0.71 [−5.18; 6.70]	2.13 [−3.10; 7.39]	**0.38** [−7.41; 8.35]	-0.65 [−9.54; 8.54]	0.87 [−6.41; 8.64]
Saturated Fatty Acids, E%	P-Alb	-2.58 [−9.85; 4.58]	**-3.39** [−10.30; 3.85]	-4.07 [−11.26; 3.42]	-2.80 [−9.88; 5.06]	**-2.23** [−9.00; 4.21]	-2.80 [−9.46; 4.27]	-1.17 [−7.44; 4.97]	**-2.19** [−9.54; 5.17]	-2.48 [−9.24; 5.09]	-1.63 [−9.61; 5.69]
Hydroxycholecalciferol	P-Alb	1.71 [−6.25; 10.13]	**2.12** [−5.96; 12.13]	1.61 [−6.54; 10.42]	3.09 [−5.98; 13.35]	**0.90** [−5.30; 7.19]	0.54 [−6.56; 8.47]	1.68 [−6.08; 9.36]	**3.16** [−6.04; 13.07]	2.24 [−7.66; 12.55]	3.47 [−6.70; 13.94]
Polyunsaturated Fatty Acids, E%	P-Alb	-0.93 [−6.18; 5.86]	**-1.21** [−5.89; 3.58]	-1.43 [−6.40; 4.03]	-0.89 [−5.79; 4.35]	**-2.05** [−5.86; 1.40]	-2.42 [−6.01; 1.27]	-1.64 [−5.96; 2.41]	**-1.17** [−5.52; 3.44]	-1.27 [−6.31; 3.49]	-0.92 [−5.68; 3.78]
Potassium	P-Alb	-3.14 [−15.40; 4.12]	**-3.89** [−9.99; 1.30]	-5.04 [−12.20; 0.79]	-3.63 [−9.67; 2.06]	**-1.06** [−4.53; 1.96]	-1.97 [−8.76; 2.01]	0.34 [−3.64; 3.96]	**-0.24** [−5.53; 5.54]	-1.02 [−7.03; 5.17]	0.27 [−5.92; 8.05]
Renavit	P-Alb	10.04 [−12.10; 24.48]	**11.73** [−8.72; 25.68]	10.55 [−8.97; 24.74]	12.77 [−7.93; 28.05]	**9.33** [−9.77; 24.03]	7.75 [−10.19; 22.86]	10.62 [−10.79; 25.61]	**8.25** [−12.70; 22.49]	7.28 [−12.78; 21.69]	9.12 [−12.99; 23.78]
Fat E%	P-Alb	3.19 [−14.81; 19.59]	**2.32** [−16.46; 20.22]	1.52 [−17.42; 20.12]	3.00 [−15.81; 20.48]	**3.40** [−12.94; 18.61]	2.52 [−15.43; 17.92]	4.14 [−12.29; 19.78]	**3.50** [−14.06; 19.88]	2.88 [−14.63; 19.85]	3.95 [−14.63; 21.22]
Phosphorous	P-Alb	-0.19 [−7.16; 6.56]	**-2.10** [−10.73; 5.28]	-3.09 [−12.68; 4.94]	-1.87 [−12.44; 7.81]	**0.27** [−5.57; 5.95]	-0.41 [−6.77; 6.08]	1.73 [−4.98; 8.63]	**0.80** [−7.31; 8.48]	0.10 [−8.68; 8.11]	1.22 [−6.42; 8.81]
Protein, g/kg	P-Alb	0.33 [−12.64; 13.11]	**-1.35** [−16.07; 11.86]	-2.72 [−17.16; 10.61]	-0.19 [−15.66; 14.04]	**-0.28** [−11.68; 11.80]	-0.95 [−12.82; 11.39]	0.98 [−11.00; 15.10]	**1.09** [−13.08; 14.12]	0.74 [−12.95; 13.72]	1.62 [−12.93; 15.03]
Vitamin D	P-Alb	1.60 [−1.28; 4.01]	**1.55** [−1.01; 4.27]	0.79 [−3.52; 4.84]	2.39 [−0.82; 6.41]	**0.79** [−1.25; 2.87]	0.11 [−3.45; 3.32]	1.45 [−1.76; 4.78]	**1.50** [−1.49; 4.58]	1.24 [−2.88; 5.29]	2.22 [−1.24; 6.60]
Fiber	P-Alb	1.19 [−5.02; 7.55]	**1.43** [−2.36; 5.60]	1.00 [−3.40; 6.23]	2.02 [−2.56; 7.51]	**1.34** [−1.91; 4.70]	0.70 [−3.35; 4.61]	2.01 [−2.06; 6.49]	**1.60** [−3.85; 6.61]	1.28 [−4.24; 6.85]	1.92 [−4.02; 7.59]
Diabetes medication	P-Alb	0.67 [−6.75; 8.48]	**0.45** [−6.07; 8.60]	-0.22 [−8.31; 8.33]	1.58 [−5.59; 9.25]	**-0.12** [−6.22; 5.63]	-0.98 [−7.54; 5.88]	1.55 [−6.13; 11.84]	**0.49** [−7.22; 8.87]	0.03 [−9.63; 9.68]	1.40 [−8.00; 11.06]
Phosphate binder med.	P-K	0.28 [−1.55; 2.75]	**0.25** [−1.83; 2.88]	-0.21 [−2.39; 2.53]	0.61 [−2.15; 3.88]	**0.40** [−1.23; 2.46]	0.04 [−2.01; 2.50]	0.68 [−1.49; 3.63]	**0.26** [−1.45; 2.40]	-0.14 [−2.83; 2.70]	0.70 [−1.44; 3.12]
Phosphate binder med.	fP-Pi	0.50 [−1.50; 2.94]	**0.61** [−0.97; 2.71]	0.33 [−2.11; 2.69]	0.82 [−1.44; 3.28]	**0.41** [−0.90; 2.00]	0.15 [−1.86; 1.99]	0.73 [−1.34; 3.02]	**0.12** [−1.71; 1.81]	-0.05 [−2.23; 1.87]	0.18 [−1.91; 2.04]
Protein, E%	P-Alb	0.64 [−7.30; 7.41]	**2.08** [−4.84; 8.78]	1.70 [−5.21; 8.26]	2.67 [−4.80; 10.24]	**1.72** [−5.00; 8.28]	1.17 [−5.17; 7.86]	2.40 [−5.78; 9.59]	**1.27** [−5.70; 8.44]	0.88 [−7.16; 8.59]	1.78 [−5.68; 8.87]
Protein, g/kg	P-K	-0.22 [−2.93; 2.74]	**0.09** [−2.42; 2.57]	-0.06 [−2.63; 2.54]	0.21 [−3.09; 2.96]	**0.03** [−2.50; 2.41]	-0.22 [−3.16; 2.65]	0.18 [−2.37; 2.65]	**-0.33** [−3.63; 2.99]	-0.36 [−3.95; 3.03]	-0.25 [−3.63; 3.12]
Carbohydrates, E%	P-K	1.05 [−2.32; 4.69]	**1.26** [−2.36; 4.61]	1.12 [−2.64; 4.81]	1.32 [−2.46; 4.82]	**0.85** [−2.16; 3.43]	0.67 [−2.37; 3.40]	0.93 [−2.40; 3.47]	**0.74** [−2.67; 4.07]	0.68 [−2.99; 4.04]	0.83 [−2.73; 4.47]
Calcium	P-K	-0.09 [−1.49; 1.54]	**-0.46** [−2.59; 0.97]	-0.63 [−3.83; 1.03]	-0.36 [−2.93; 1.41]	**0.01** [−0.94; 1.17]	-0.15 [−1.92; 1.29]	0.38 [−0.76; 2.19]	**-0.13** [−1.62; 1.29]	-0.37 [−2.41; 1.41]	0.05 [−1.49; 1.56]
Energy, kcal/kg	P-K	0.30 [−2.53; 4.98]	**-0.19** [−2.92; 2.96]	-0.30 [−3.37; 2.87]	-0.11 [−2.54; 2.94]	**-0.14** [−2.52; 2.41]	-0.24 [−2.71; 2.37]	-0.09 [−2.64; 2.53]	**0.29** [−3.70; 4.70]	0.17 [−3.81; 4.57]	0.43 [−3.70; 4.95]
Salt	P-K	0.11 [−1.82; 2.50]	**0.93** [−1.37; 2.94]	0.74 [−1.96; 2.72]	1.09 [−1.35; 3.42]	**0.18** [−0.79; 1.10]	-0.09 [−2.57; 1.23]	0.38 [−0.64; 1.41]	**-0.24** [−2.89; 1.63]	-0.50 [−3.94; 1.64]	-0.20 [−3.15; 1.68]
Gender	P-K	-0.04 [−2.53; 2.63]	**0.41** [−1.36; 2.47]	0.35 [−1.54; 2.50]	0.57 [−1.36; 2.75]	**0.26** [−1.17; 1.78]	0.07 [−1.57; 1.95]	0.39 [−1.40; 2.49]	**0.92** [−1.97; 4.50]	0.76 [−2.39; 4.48]	1.07 [−1.90; 4.28]
Blood lipid medication	P-K	-0.65 [−3.47; 1.82]	**0.11** [−2.42; 4.45]	-0.21 [−3.88; 4.27]	0.36 [−2.46; 5.41]	**-0.32** [−1.68; 0.98]	-0.61 [−2.45; 0.86]	0.10 [−1.30; 1.86]	**-0.55** [−3.45; 1.73]	-0.96 [−5.15; 1.91]	-0.23 [−3.25; 3.33]

Nutrient	Conc.	General effect	Home hemodialysis			Hospital hemodialysis			Peritoneal dialysis		
			avg	min	max	avg	min	max	avg	min	max
Carbohydrates, E%	fP-Pi	0.29 [−2.56; 2.87]	**0.00** [−1.88; 1.73]	-0.07 [−1.88; 1.68]	0.03 [−1.82; 1.79]	**0.16** [−1.40; 1.58]	0.08 [−1.66; 1.53]	0.27 [−1.08; 1.59]	**0.06** [−1.90; 1.87]	0.01 [−1.89; 1.85]	0.08 [−1.99; 1.97]
Monounsaturated Fatty Acids, E%	P-K	0.43 [−1.45; 3.02]	**0.76** [−0.85; 3.37]	0.72 [−0.93; 3.46]	0.93 [−0.87; 3.74]	**0.52** [−0.75; 2.02]	0.28 [−1.18; 1.77]	0.66 [−0.96; 3.02]	**0.58** [−1.48; 2.81]	0.45 [−1.80; 2.80]	0.72 [−1.20; 2.68]
Sodium	fP-Pi	0.19 [−1.69; 1.93]	**0.63** [−0.91; 3.46]	0.57 [−0.85; 3.26]	0.70 [−0.86; 3.47]	**0.12** [−0.65; 0.72]	0.05 [−0.76; 0.68]	0.20 [−0.57; 0.85]	**0.41** [−1.65; 3.99]	0.36 [−1.60; 3.80]	0.47 [−1.68; 4.15]
Sodium	P-K	-0.15 [−1.98; 2.35]	**-0.06** [−1.88; 2.31]	-0.15 [−1.98; 2.23]	-0.02 [−1.88; 2.32]	**-0.25** [−1.74; 1.63]	-0.40 [−1.86; 1.45]	-0.05 [−1.76; 2.26]	**-0.54** [−2.64; 2.16]	-0.60 [−2.74; 2.22]	-0.39 [−2.55; 2.47]
Potassium	P-K	-0.46 [−3.35; 2.02]	**-0.06** [−1.48; 1.55]	-0.32 [−2.14; 1.32]	0.14 [−1.57; 1.76]	**0.14** [−0.58; 1.02]	-0.11 [−1.40; 1.27]	0.50 [−0.52; 1.64]	**-0.21** [−1.66; 0.98]	-0.51 [−2.22; 1.17]	0.16 [−1.50; 2.45]
Diabetes medication	fP-Pi	0.20 [−0.64; 1.86]	**0.19** [−0.71; 1.33]	0.04 [−1.14; 1.37]	0.37 [−0.74; 1.72]	**0.05** [−0.68; 0.77]	-0.09 [−1.12; 0.90]	0.10 [−0.76; 1.09]	**0.17** [−0.95; 1.93]	0.04 [−1.28; 2.04]	0.33 [−1.12; 2.12]
Polyunsaturated Fatty Acids, E%	fP-Pi	0.22 [−1.14; 2.26]	**-0.52** [−1.63; 0.31]	-0.53 [−1.81; 0.37]	-0.50 [−1.62; 0.40]	**-0.34** [−0.89; 0.09]	-0.40 [−1.04; 0.10]	-0.29 [−0.80; 0.16]	**-0.25** [−0.97; 0.62]	-0.28 [−1.06; 0.64]	-0.21 [−0.93; 0.64]
Blood lipid medication	fP-Pi	0.02 [−1.88; 3.27]	**-0.81** [−3.36; 1.66]	-0.95 [−3.36; 2.11]	-0.67 [−3.58; 1.79]	**-0.51** [−1.24; 0.19]	-0.82 [−2.45; 0.29]	-0.33 [−1.19; 0.49]	**-0.14** [−1.96; 2.11]	-0.33 [−2.22; 1.70]	-0.07 [−2.23; 2.54]
Diabetes medication	P-K	5.41 [−1.85; 23.43]	**-0.17** [−1.92; 2.00]	-0.35 [−2.73; 2.46]	0.07 [−1.90; 2.35]	**-0.13** [−1.29; 1.14]	-0.31 [−2.35; 1.47]	0.08 [−1.18; 1.81]	**-0.65** [−4.90; 2.38]	-0.76 [−4.82; 2.23]	-0.54 [−5.11; 2.94]
Energy, kcal/kg	fP-Pi	0.50 [−1.50; 2.17]	**0.58** [−0.90; 2.05]	0.46 [−0.95; 1.93]	0.68 [−0.90; 2.25]	**0.71** [−0.72; 1.96]	0.63 [−0.76; 1.91]	0.80 [−0.72; 2.08]	**0.51** [−1.79; 2.22]	0.45 [−1.83; 2.21]	0.56 [−1.88; 2.41]
Fat E%	fP-Pi	0.21 [−1.81; 2.17]	**0.51** [−1.48; 2.46]	0.46 [−1.50; 2.43]	0.57 [−1.46; 2.74]	**0.57** [−1.22; 2.58]	0.50 [−1.26; 2.55]	0.63 [−1.25; 2.79]	**0.18** [−1.91; 2.15]	0.13 [−1.82; 2.12]	0.24 [−2.00; 2.17]
Renavit	P-K	0.94 [−3.25; 7.50]	**0.29** [−3.83; 6.53]	0.11 [−4.16; 6.46]	0.58 [−3.46; 6.62]	**0.76** [−2.53; 5.74]	0.49 [−3.01; 5.34]	0.93 [−2.38; 5.89]	**0.88** [−3.09; 6.47]	0.92 [−3.27; 6.59]	1.08 [−2.84; 6.36]
Water	P-K	0.16 [−0.99; 1.79]	**0.18** [−0.59; 1.08]	-0.04 [−1.81; 1.35]	0.36 [−0.54; 1.31]	**-0.24** [−0.92; 0.44]	-0.48 [−1.67; 0.65]	0.01 [−0.69; 0.56]	**-0.10** [−1.53; 1.13]	-0.33 [−1.93; 1.27]	0.01 [−1.53; 1.54]
Water	fP-Pi	0.11 [−0.71; 0.73]	**0.14** [−0.29; 0.60]	0.03 [−0.88; 0.69]	0.24 [−0.37; 0.89]	**0.17** [−0.13; 0.55]	0.00 [−0.30; 0.34]	0.23 [−0.20; 0.87]	**0.05** [−0.81; 0.99]	-0.08 [−1.18; 0.97]	0.21 [−0.69; 1.08]
Fiber	P-K	-0.92 [−6.53; 1.42]	**0.23** [−0.72; 1.48]	-0.07 [−1.45; 1.17]	0.46 [−0.85; 1.85]	**0.03** [−0.93; 0.94]	-0.29 [−1.45; 0.96]	0.28 [−1.04; 1.33]	**0.47** [−1.04; 2.48]	0.31 [−1.32; 2.59]	0.62 [−1.21; 2.79]
Renavit	fP-Pi	0.14 [−2.13; 3.06]	**0.36** [−2.03; 2.93]	0.28 [−2.04; 2.94]	0.39 [−2.03; 2.96]	**0.20** [−1.60; 1.78]	0.14 [−1.73; 1.80]	0.26 [−1.63; 1.89]	**0.77** [−2.49; 4.08]	0.70 [−2.59; 3.99]	0.83 [−2.47; 4.02]
Vitamin D	P-K	-0.17 [−1.20; 1.11]	**-0.15** [−0.94; 0.60]	-0.25 [−1.23; 0.78]	-0.11 [−0.91; 0.79]	**-0.18** [−0.65; 0.45]	-0.33 [−1.04; 0.46]	0.02 [−0.44; 0.60]	**-0.31** [−1.24; 0.72]	-0.49 [−1.32; 0.42]	-0.22 [−1.21; 0.95]
Protein, g/kg	fP-Pi	-0.80 [−2.41; 1.10]	**-0.84** [−2.37; 0.91]	-0.91 [−2.49; 0.86]	-0.78 [−2.52; 0.99]	**-0.81** [−2.03; 0.65]	-0.86 [−2.16; 0.57]	-0.74 [−2.03; 0.74]	**-0.64** [−2.32; 1.06]	-0.65 [−2.29; 1.10]	-0.60 [−2.38; 1.12]
Fat E%	P-K	-0.38 [−4.54; 3.29]	**-0.14** [−4.80; 3.86]	-0.26 [−5.16; 3.77]	-0.06 [−4.85; 4.02]	**-0.25** [−4.41; 3.08]	-0.40 [−4.67; 2.98]	-0.01 [−4.12; 3.17]	**-0.51** [−5.07; 3.44]	-0.65 [−5.50; 3.48]	-0.41 [−4.82; 3.39]
Protein, E%	fP-Pi	0.20 [−1.06; 1.87]	**0.12** [−0.65; 0.79]	0.08 [−0.70; 0.85]	0.16 [−0.68; 0.81]	**0.24** [−0.49; 0.84]	0.20 [−0.56; 0.87]	0.30 [−0.39; 0.92]	**0.14** [−1.03; 1.09]	0.09 [−1.04; 1.05]	0.17 [−1.05; 1.16]
Monounsaturated Fatty Acids, E%	fP-Pi	-0.10 [−1.84; 1.77]	**0.06** [−0.73; 1.05]	0.03 [−0.80; 1.06]	0.12 [−0.77; 1.21]	**-0.13** [−0.89; 0.62]	-0.16 [−1.04; 0.63]	-0.05 [−0.88; 0.72]	**-0.02** [−0.95; 0.94]	-0.06 [−1.01; 0.97]	0.02 [−0.91; 1.00]
Polyunsaturated Fatty Acids, E%	P-K	0.29 [−1.25; 1.84]	**0.28** [−1.11; 1.73]	0.21 [−1.28; 1.85]	0.38 [−1.21; 2.24]	**0.15** [−0.71; 1.10]	0.00 [−1.02; 1.07]	0.25 [−0.76; 1.48]	**0.32** [−0.91; 1.86]	0.19 [−1.05; 1.44]	0.45 [−0.81; 2.22]
Phosphorous	P-K	0.39 [−2.48; 2.31]	**0.25** [−1.41; 2.02]	0.15 [−1.57; 1.79]	0.36 [−1.54; 2.84]	**0.21** [−1.20; 1.69]	0.10 [−1.37; 1.54]	0.37 [−1.30; 2.61]	**0.51** [−1.42; 2.55]	0.45 [−1.44; 2.39]	0.59 [−1.49; 2.61]
Protein, E%	P-K	0.49 [−1.46; 3.27]	**0.23** [−0.93; 2.24]	0.13 [−1.23; 2.20]	0.25 [−0.99; 2.24]	**0.28** [−1.23; 2.16]	0.15 [−1.46; 2.25]	0.42 [−1.03; 2.26]	**0.54** [−1.25; 3.15]	0.46 [−1.49; 3.14]	0.52 [−1.32; 3.09]
Saturated Fatty Acids, E%	P-K	1.01 [−1.10; 3.03]	**0.86** [−1.48; 3.10]	0.77 [−1.34; 2.93]	0.95 [−1.50; 3.18]	**0.82** [−0.95; 2.82]	0.64 [−1.47; 2.69]	0.95 [−0.86; 3.08]	**1.06** [−1.01; 3.19]	1.01 [−1.17; 3.28]	1.18 [−0.85; 3.25]
Saturated Fatty Acids, E%	fP-Pi	-0.36 [−1.51; 0.79]	**-0.53** [−2.02; 0.56]	-0.58 [−2.04; 0.57]	-0.49 [−2.07; 0.67]	**-0.32** [−1.22; 0.50]	-0.39 [−1.34; 0.50]	-0.27 [−1.24; 0.65]	**-0.05** [−1.14; 0.96]	-0.06 [−1.19; 0.98]	-0.01 [−1.15; 1.03]
Fiber	fP-Pi	-0.21 [−1.69; 1.06]	**-0.11** [−0.60; 0.37]	-0.18 [−0.79; 0.36]	-0.08 [−0.56; 0.41]	**-0.11** [−0.66; 0.30]	-0.19 [−0.79; 0.21]	-0.05 [−0.69; 0.56]	**-0.13** [−1.11; 0.96]	-0.16 [−1.25; 0.97]	-0.09 [−1.15; 1.09]
Calcium	fP-Pi	-0.25 [−1.24; 0.42]	**-0.04** [−0.54; 0.56]	-0.11 [−0.93; 0.69]	0.17 [−0.63; 1.12]	**-0.15** [−0.59; 0.22]	-0.30 [−1.19; 0.29]	0.01 [−0.56; 0.66]	**-0.06** [−0.96; 0.74]	-0.24 [−1.12; 0.84]	0.00 [−1.04; 1.04]
Hydroxycholecalciferol	fP-Pi	1.51 [−1.59; 7.33]	**0.52** [−2.27; 3.05]	0.40 [−2.44; 3.08]	0.70 [−2.10; 3.25]	**0.49** [−0.30; 1.38]	0.40 [−0.64; 1.38]	0.59 [−0.42; 1.77]	**0.17** [−2.04; 1.54]	-0.05 [−2.09; 1.56]	0.26 [−2.08; 1.65]
Gender	fP-Pi	-0.13 [−1.66; 0.88]	**-0.02** [−1.32; 1.06]	-0.14 [−1.51; 1.14]	0.06 [−1.46; 1.22]	**-0.14** [−1.12; 0.76]	-0.28 [−1.42; 0.95]	0.05 [−1.04; 1.10]	**-0.04** [−1.34; 1.14]	-0.07 [−1.41; 1.22]	0.02 [−1.53; 1.54]
Vitamin D	fP-Pi	0.10 [−0.37; 0.54]	**0.06** [−0.41; 0.58]	-0.07 [−0.69; 0.46]	0.13 [−0.54; 1.01]	**0.15** [−0.14; 0.41]	0.05 [−0.44; 0.52]	0.29 [−0.14; 0.71]	**0.14** [−0.40; 0.64]	0.16 [−0.52; 0.77]	0.22 [−0.35; 0.82]
Potassium	fP-Pi	0.07 [−0.88; 0.88]	**0.00** [−0.72; 0.76]	-0.05 [−0.82; 0.77]	0.04 [−0.72; 0.78]	**0.06** [−0.34; 0.52]	0.03 [−0.51; 0.53]	0.09 [−0.35; 0.56]	**0.17** [−0.56; 0.97]	0.15 [−0.68; 1.03]	0.21 [−0.48; 1.05]
Phosphorous	fP-Pi	0.08 [−0.90; 1.41]	**0.15** [−1.00; 1.13]	0.10 [−1.19; 1.23]	0.21 [−1.14; 1.21]	**-0.02** [−0.79; 0.79]	-0.09 [−0.95; 0.81]	0.08 [−0.63; 0.76]	**0.16** [−0.91; 1.43]	0.05 [−1.22; 1.43]	0.20 [−0.90; 1.45]
Salt	fP-Pi	0.61 [−1.26; 3.65]	**-0.66** [−3.44; 0.90]	-0.71 [−3.64; 0.86]	-0.62 [−3.37; 0.96]	**-0.11** [−0.52; 0.39]	-0.16 [−0.91; 0.42]	-0.04 [−0.54; 0.56]	**-0.66** [−3.86; 1.21]	-0.73 [−4.08; 1.25]	-0.64 [−3.74; 1.22]
