# Supplementary material for: Inferring personal intake recommendations of phosphorous and potassium for end-stage renal failure patients by simulating with Bayesian hierarchical multivariate model
Source: PLoS One. 2024 Feb 6;19(2):e0291153. doi: 10.1371/journal.pone.0291153 (PMC10846746; doi:10.1371/journal.pone.0291153)
Supplement: S4 Table — (PDF) [file pone.0291153.s009.pdf]

Table S4: Notation summary of the inference method

Notation	Description
b	Personal effect, in addition to treatment effect
c	Constant for shifting the distribution
g	Treatment effect, in addition to the general effect
G_k	Personal graphical model for patient k
G_k^*	Personal graphical model with modified intake
i	Observation index
j	Predictor index (nutrient and medication)
J	Number of predictors
k	Patient index
K	Number of patients
l	Dialysis treatment type index
L	Maximum number of treatments
l_x	Quantile of intake random variable used in the estimation
l_β	Quantile of nutrient effect used in the estimation
m	Concentration index
M	Number of considered concentrations
n	Number of observations
p	Number of predictors
P_m^{max}	Maximum probability for reaching target for concentration m
P^{max}	Maximum probability for reaching all the concentration targets
Q	Recommended nutrient
Q_r^{min}	Lower limit of 95%-quantile value for recommended nutrient r
Q_r^{max}	Upper limit of 95%-quantile value for recommended nutrient r
r	Recommended nutrient index
R	Number of recommended nutrients
s	Recommendation algorithm sample index
S	Number of samples to draw from the recommendation algorithm
Y	Concentration level
Y_m^l	Lower limit of concentration m
Y_m^u	Upper limit of concentration m
X	General predictor, intake level or personal information
$\mathbf{X}$	p x n matrix of predictor observations
Z	Personally varying predictor, intake level or personal information
$\mathbf{Z}$	p x n matrix of predictor observations
α	Shape parameter of gamma distribution
β_{jm}	General effect of predictor j to concentration m
μ_m	Expected value of concentration m
ρ_{mn}	Correlation between random variables m and n
$\otimes$	Kronecker product
