# Supplementary material for: Inferring personal intake recommendations of phosphorous and potassium for end-stage renal failure patients by simulating with Bayesian hierarchical multivariate model
Source: PLoS One. 2024 Feb 6;19(2):e0291153. doi: 10.1371/journal.pone.0291153 (PMC10846746; doi:10.1371/journal.pone.0291153)
Supplement: Attachment — Submitted filename: Response to Reviewers.pdf [file pone.0291153.s010.pdf]

This document describes a point-by-point response to reviewers for a PLOS ONE submitted article “Inferring personal intake recommendations of phosphorous and potassium for end-stage renal failure patients by simulating with Bayesian hierarchical multivariate model”

Ref: PONE-D-23-20128

Authors: Jari Turkia, Ursula Schwab, and Ville Hautamäki

Dear Editor,

Thank you for the review and valuable comments. We have acknowledged your concerns about the paper requiring too much statistical background information and have done our best to clarify the writing and figures for the paper to be most valuable to readers. We have addressed your concerns point-by-point as follows.

Journal Requirements:

1. Please ensure that your manuscript meets PLOS ONE's style requirements, including those for file naming.

Response > We have ensured that the manuscript follows these style requirements:

https://journals.plos.org/plosone/s/file?id=wjVg/PLOSOne_formatting_sample_main_body.pdf
https://journals.plos.org/plosone/s/file?id=ba62/PLOSOne_formatting_sample_title_authors_affiliations.pdf

In this revision we have made following stylistic changes:

- Corresponding author is denoted as in the guide:

  * Corresponding author
  E-mail: jari.turkia@cgi.com (JT)

- File naming is according to the requirements.

2. In your Data Availability statement, you have not specified where the minimal data set underlying the results described in your manuscript can be found. PLOS defines a study's minimal data set as the underlying data used to reach the conclusions drawn in the manuscript and any additional data required to replicate the reported study findings in their entirety. All PLOS journals require that the minimal data set be made fully available. For more information about our data policy, please see http://journals.plos.org/plosone/s/data-availability.

Upon re-submitting your revised manuscript, please upload your study's minimal underlying data set as either Supporting Information files or to a stable, public repository and include the relevant URLs, DOIs, or accession numbers within your revised cover letter. For a list of acceptable repositories, please see http://journals.plos.org/plosone/s/data-availability#loc-recommended-repositories. Any potentially identifying patient information must be fully anonymized.

Response > The minimal data set has been available in a Github repository so that it works with the supplementary source code, and we have now uploaded the minimal data set also in the Supporting Information files for easier access.

3. Please include your full ethics statement in the 'Methods' section of your manuscript file. In your statement, please include the full name of the IRB or ethics committee who approved or waived your study, as well as whether or not you obtained informed written or verbal consent. If consent was waived for your study, please include this information in your statement as well.

Response> We have added details about the IRB, Kuopio University Hospital Research Assistance Center ("KYS Tiedepalvelukeskus" in Finnish), and the waived ethics approval to the Dialysis patient data section. The section also mentions the written informed consent that was obtained.

Reviewer 1

In terms of personalized medicine an approach to adjust dietary recommendations is highly interesting, especially for the management of potassium, phosphate and protein in dialysis patients, which is still a big issue in nephrology. However, the provided manuscript in its current version might be difficult to understand for the medical community and requires modifications.

This accounts especially for the graphical models with additional explanations and more guidance is needed how to apply the models on the personalized diet recommendations.

Most figure designs are quite challenging and should be simplified and structured more clearly to address the readers.

1. **How were the variables selected that were implemented in the Bayesian model (Serum concentrations and nutrient predictors)? This is especially of interest as, also stated by the authors, there is only limited direct associations between phosphate, protein and potassium.**

   Response> The aim of the analysis was to explore the possibility of less restricted phosphorous and potassium intake while making sure that their concentrations stayed in their normal ranges. All the other nutrient predictors in the model were selected to reflect patients' current diet. We assumed all the energy nutrients, vitamins, minerals, and fluids to

be relevant in the determining the concentrations. Possible similar effects of nutrients (collinearity) were mitigated with QR-decomposition in the model. This is now clarified in the data section:

"Laboratory tests for renal patients included several measurements, from which concentrations of plasma potassium (P-K), fasting plasma phosphorous (fP-Pi), and plasma albumin (P-Alb) were selected as targets of this analysis for exploring the possibility of less restricted phosphorous and potassium intake. The selected predictors were assumed to reflect the composition of these concentrations; all the energy nutrients, vitamin D, minerals, and fluids. Also, the selected medications were known to directly affect the concentrations. Patients fasted before the laboratory tests although there were analyses that did not require fasting. The schema for data collection is outlined in Fig. 1."

2. **How was the time interval between blood sampling and dialysis for both blood collections? Dialysis treatment will adjust for serum potassium and in parts for serum phosphate. A small scheme depicting the workflow, and including the interview parts would be helpful.**

   Response> The laboratory tests always occurred before dialysis treatment. This is now clarified in the text. We appreciate the suggested scheme for data collection workflow, and it is now added in the revision.

3. **For phosphorous it is mentioned that patients were fasted for potassium and for albumin not (which would be preferred)?- Does that mean there were different sampling timepoints for each visit? And if so, what was eaten in between?**

   Response> All the analyses were taken at the same timepoints, and patients fasted before the laboratory tests, but for example, P-Alb does not require fasting, so it is not marked in the notation. Fasting is also clarified now in the text and noted in the workflow scheme.

4. **Not all parameters/ abbreviations seem to be explained in the description of the personalized graphical models and the unrelated regressions. These sections are very difficult to read and should be rephrased. A table explaining all variables (e.g. as supplementary table) could help.**

   Response> We have elaborated the key concepts in this section more carefully and made sure that all the variables and notations are explained in the text. The text is also revised for better clarity. Supplementary Table S4 has also been added to summarize the variables and other notations of the method section.

5. **I appreciate the idea showing a personalized recommendation for one single patient (figure 2). However the given association with albumin is difficult to understand. The 3 small graphs are quite confusing- if intakes and serum levels are depicted in one graph, this needs to be stated more clearly. Should dietary protein be considered here? The benefit of the model for the personalized recommendation should be worked out more clearly (e.g. if the patient increases intake of x, and increase in y becomes more likely..) and the graph requires a more clear structure and explanations of the color code.**

   Response> In Figures 2 and 3, one key aspect is to show the recommended nutrient ranges and the concentrations that the recommendations are predicted to produce. Keeping them both in the same Figures aims to show the function of our method. We clarified the figures in this revision and added explanations of the color coding that binds these subfigures.

   Dietary protein would possibly affect the modeled concentrations, but in this study, we chose to consider only phosphorous and potassium intakes and the possibility of less restricted intake. As stated in Discussion, modifying these nutrients alone is not enough for every patient, and in our future work our aim is to generalize the present method to modify the whole diet. Biological interpretation of the model, in context of Fig. 2, is also worked out in the Discussion section. In this revision, we have emphasized in Fig 4 the patients who could be recommended a less restrictive diet. This is the main benefit of the model.

6. **Table 5 seems very relevant and could help to improve therapeutical options. But why was the intrapersonal effect versus the inter-patient variation shown and in supplementary table 3 it was stratified by type of dialysis? The general effect should be implemented into the main figure.**

   Response> We have added the general effect to Table 5 and removed the intra-patient variation as becomes clear from the already presented minimum and maximum of personal effects. Treatment induced variation is still shown as due to different principles of action it affects the nutrient effects and is a relevant predictor.

7. **What do the green marks in figure 3 indicate? Is this the recommended range per parameter? Why are ranges (first columns) and recommendations (column 3-5) mixed in one graph? Again, quite confusing to a reader. Why not showing recommended daily nutrient intakes as well?**

   Response> The green marks in the potassium and phosphorous columns indicate the personally recommended range of intake, and the numbers in those columns indicate the 90%-quantile values of the range. We agree that the Figure is busy and we have now removed some unnecessary information like confidence percentages of predictions. The intake columns already show the general recommendations of potassium and phosphorous intake (with solid black lines), and the green marks have been changed to blue and red

color-coding to indicate if personal recommendation exceeds the general recommendation. This is the key takeaway from this Figure.

It is essential to keep the intake recommendations and the corresponding concentration predictions in the same Figure as the concentration predictions induce the recommendations. Splitting these in separate Figures would make this connection hard to trace. We have added more legends to the Figure and rephrased the caption to make this connection clearer.

8. **Figure 4: The descriptions in the posterior predictive check are too complicated- what is the observed data and what is the predicted one (black line? Purple line?)**

   Response> We have simplified the caption for the posterior predictive check: “Posterior Predictive Check (PPC) for the final model version: Black lines represent observed concentration levels, while purple lines overlay the predicted concentration levels when all predictor nutrients match observed values. For an unbiased model, the observed and predicted concentrations are expected to be aligned.”

Sincerely,

Mr. Jari Turkia,
Corresponding Author

On behalf of all the authors
